# Impact of a pandemic on surgical neuro-oncology—maintaining functionality in the early phase of crisis

**DOI:** 10.1186/s12893-021-01055-z

**Published:** 2021-01-18

**Authors:** N. U. Farrukh Hameed, Yixin Ma, Zili Zhen, Shuai Wu, Rui Feng, Weiping Li, Guodong Huang, Jinsong Wu, Zhongping Chen

**Affiliations:** 1 Department of Neurosurgery, Huashan Hospital, Fudan University, Shanghai, 201206 China; 2 Department of General Surgery, Jinshan Hospital, Fudan University, Shanghai, 201206 China; 3 Department of Neurosurgery, The First Affiliated Hospital of Shenzhen University, Shenzhen, 518035 China

**Keywords:** COVID-19, Pandemic, Neuro-oncological surgery, Neurosurgery, Neuro-oncology

## Abstract

**Background:**

The Coronavirus disease 2019 (COVID-19) pandemic has extraordinarily impacted global healthcare. Neuro-oncological surgery units have peculiar features that make them highly relevant in the strategic reaction to the pandemic. In this Chinese Society of Neuro-Oncology (CSNO) initiated survey, we appraise the changes implemented in neuro-oncological surgery hospitals across different Asian countries and provide expert recommendations for responses at different stages of the pandemic.

**Methods:**

We performed a 42-question survey of the early experience of neuro-oncological surgery practice in hospitals across different Asian countries on April 1, 2020, with responses closed on April 18, 2020.

**Results:**

144 hospitals completed the questionnaire. Most were in WHO post-peak phase of the pandemic and reported a median reduction in neuro-oncological surgery volume of 25–50%. Most (67.4%) resumed elective surgery in only COVID-19 negative patients;11.1% performed only emergency cases irrespective of COVID-19 status;2.1% suspended all surgical activity. Ninety-one (63.2%) relocated personnel from neurosurgery to other departments. Fifty-two (36.1%) hospitals suspended post-operative adjuvant therapy and 94 (65.2%) instituted different measures to administer post-operative adjuvant therapy. Majority (59.0%) of the hospitals suspended research activity. Most (70%) respondents anticipate that current neurosurgery restrictions will continue to remain for > 1 month.

**Conclusions:**

Majority of the respondents to our survey reported reduced neuro-oncological surgery activity, policy modification, personnel reallocation, and curtailment of educational/research activities in response to the COVID-19 pandemic. The persistent widespread interruption of surgical neuro-oncology in even post-peak phases of the pandemic raises serious concerns about the long-term impact of the pandemic on neuro-oncological patients and highlights the essence of timely measures for pandemic preparedness, patient triage, and workforce protection.

## Background

The Coronavirus disease 2019 (COVID-19) pandemic has extraordinarily impacted healthcare systems on a global scale, Including the practice of neuro-oncological surgery [1–3].

Unlike most other surgical specialties, neuro-oncological surgery is uniquely associated with prolonged use of intensive care unit beds and specialized healthcare professionals to manage patients at different stages of recovery. The extensive surge in demand for prolonged ventilator use, spacing, personal protective equipment (PPE) and the reallocation of neuro-oncological surgery personnel in response to the COVID-19 pandemic has obstructed routine practice of neuro-oncological surgery internationally. Different measures have been adopted by hospitals at different phases of the pandemic to triage neurosurgery cases [4–8].

Patients with tumors of the central nervous system (CNS) present complex challenges, not only due to their immunocompromised state which increases their risk of infection, but also due to the need for extreme caution when performing surgery. Importantly, surgery cannot be deferred in such patients due to their susceptibility to infection.

In this Chinese Society of Neuro-Oncology (CSNO) initiated survey, we aimed to quantitatively evaluate the experience and adjustments implemented to date at different neuro-oncological surgery hospitals in response to the COVID-19 pandemic. By acquiring data from a broad representation of neuro-oncological surgery hospitals at different phases of the pandemic, we provide expert recommendations relating to neuro-oncological surgery planning and preferred responses at each stage of the COVID-19 pandemic and other potential infectious diseases.

## Methods

### Survey

A 42-question questionnaire was distributed by the CSNO to neuro-oncological surgery hospitals across different Asian countries on April 1, 2020 by electronic mail message. The survey was closed on April 18, 2020. One reminder message was sent to initial non-respondents and included invitations to other faculty members to join at each of the respective hospitals. Participation was voluntary and the anonymity of all respondents was preserved. The data were audited for duplicate or discordant responses. The study was approved by the Institutional Review Boards of both Huashan Hospital and The First Affiliated Hospital of Shenzhen University. The ethics committee ruled that no formal consent from participants was required for this study since it was a survey.

### Questionnaire data

The questionnaire was designed to assess the changes implemented at each hospital in response to the COVID-19 pandemic, according to the current COVID-19 status in each hospital’s jurisdiction/region, the hospital’s resources and logistics, and institutional, regional or national policies.

The questionnaire comprised 22 multiple choice questions, of which 2 used a Likert response scale. Thirteen questions contained a field for a specific numeric response, and 7 questions required free text responses. The respondents were requested to answer questions based on their current knowledge of the COVID-19 situation at their geographic location, the response of their hospitals, and their anticipated future directions. The full questionnaire is presented as Additional file 1: Table S1.

### Data analysis

Continuous variables were reported as either medians (interquartile range [IQR]) or means (standard deviation [SD]), according to visual inspection of the data and the Shapiro–Wilk normality test. Categorical variables are reported as counts and percentages.

COVID-19 infection data by country (and province/state, where available) as of April 10, 2020 were obtained from the Johns Hopkins Coronavirus Resource Center [9]. The number of infections were adjusted by population size and reported as the number of COVID-19 cases per million inhabitants. Correlations between the adjusted number of COVID-19 cases and survey variables were calculated by using Pearson correlation test for continuous variables, Spearman correlation for ordinal variables, and point-biserial correlation coefficient for dichotomous variables.

All analyses were performed by using SPSS version 22 (IBM, Chicago, IL).

## Results

### Respondent characteristics and current COVID-19 status

A total of 144 hospitals participated in the survey and completed the questionnaire (response rate of approximately 85%). The detailed locations of the hospitals and the number of COVID-19 cases per geographic region are summarized in Additional file 1: Table S2. The list of participating hospitals is presented in Additional file 1: Table S3. Majority of the respondents were surgical neuro-oncologists [72 (50.0%)] followed by general neurosurgeons [28 (19.5%)] and neuro-oncologists [20 (13.9%)] (Table 1).

**Table 1:** Status of responding hospitals in relation to the Coronavirus disease 2019 (COVID-19) pandemic

Variable	Number of hospitals (%)
Responding neurosurgeon’s specialty	
Cerebrovascular	9 (6.3)
General	28 (19.5)
Functional	5 (3.5)
Multi-specialty	20 (13.9)
Neuro-oncology	75 (52.1)
Pediatric	3 (2.1)
Skull base	1 (0.7)
Spine	3 (2.1)
First diagnosis of COVID-19 in respondents’ region	
December, 2019	8 (5.6)
January, 2020	97 (67.4)
February, 2020	33 (22.9)
March, 2020	4 (2.8)
Current number of COVID-19 cases in respondents’ region	
< 100	5 (3.5%)
101–500	1 (0.7%)
501–1000	3 (2.1%)
> 1000	135 (93.8%)
Current number of COVID-19-related deaths in respondents’ region	
< 100	0 (0%)
101–500	13 (9%)
501–1000	0 (%)
> 1000	137 (91%)
Public health restrictions in place	144 (100%)
Nature of public health restrictions	
Restrictions on public gatherings of > 100 people	1 (0.7)
Restrictions on public gatherings of > 10 people	7 (4.9)
Restrictions on all public gatherings of any size	81 (56.3)
Complete curfew/blockade, closed nonessential services	54 (37.5)
Hospital is a COVID-19 dedicated hospital	73 (50.7)
COVID-19 patients have been hospitalized at hospital	95 (66.0)
Number of COVID-19-related hospitalizations at hospital	
< 10	97 (67.4)
11–50	26 (18.1)
51–100	6 (4.2)
> 100	15 (10.4)
Percentage of COVID-19-patients by hospital capacity	
< 10%	130 (95.0)
10–50%	6 (4.3)
> 50%	1 (0.7)

All hospitals indicated that public health restrictions existed in their respective regions to limit COVID-19 spread (Table 1). A total of 73 (50.7%) hospitals were COVID-19 dedicated hospitals and 95 (66.0%) hospitals reported at least 1 COVID-19 related hospitalization. (Table 1).

### Impact of COVID-19 pandemic on neurosurgery activity

**Fig. 1:**
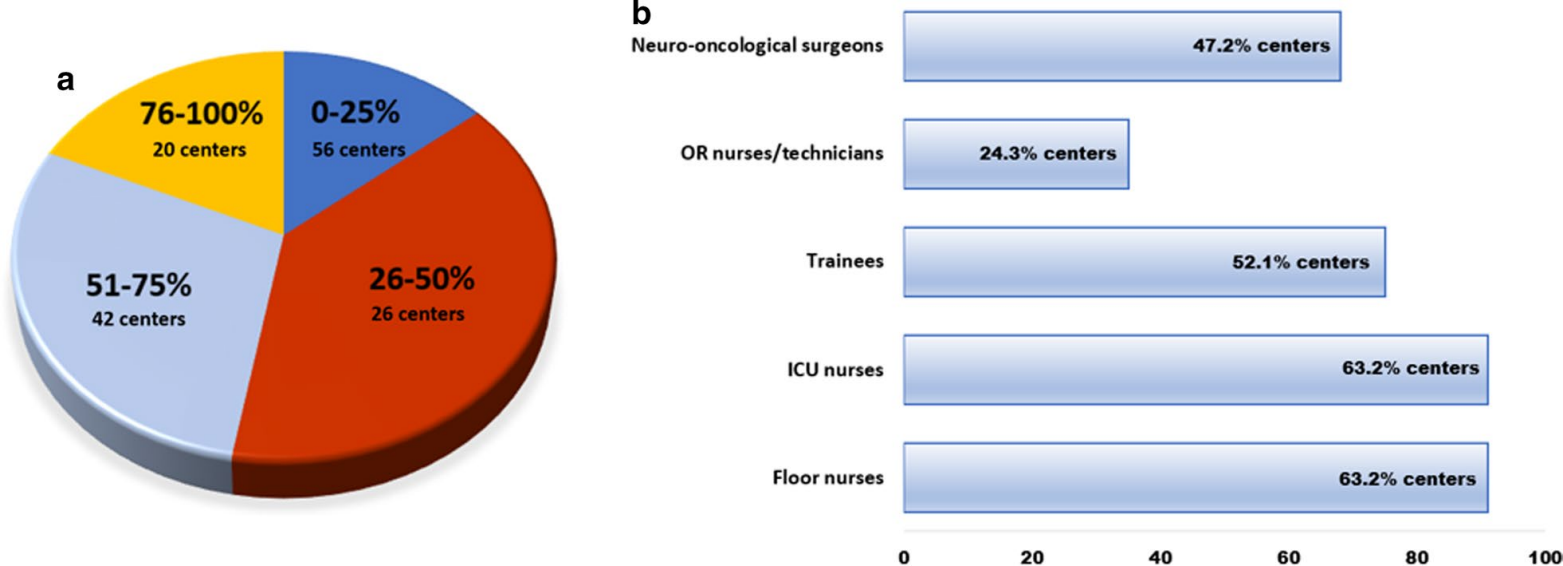
Response of centers to the Coronavirus disease 2019 (COVID-19) pandemic**.** **a** Percentage reduction in neuro-oncological surgery activity at the responding hospitals; **b** Personnel relocated from neuro-oncological surgery to other departments

The median annual neurosurgery volume and number of neurosurgery dedicated beds of the responding hospitals before the COVID-19 pandemic were 400 (IQR 350; 500) cases and 63 (IQR 45; 116) beds respectively. The median reduction in neurosurgery case volume was 25–50%. Twenty (13.9%) hospitals reported a < 25% reduction in their neurosurgery case volume due to the COVID-19 pandemic, and 26 (18.1%) reported a 76–100% reduction (Fig. 1), not correlated [correlation coefficient, r = 0.24, *p* = 0.24] with the number of COVID-19 cases per million inhabitants in the respondent’s country/province/state (Table 2).

**Table 2:** Impact of Coronavirus disease 2019 (COVID-19) pandemic on neurosurgery activity and facilities, education and research

Variable	Number of hospitals (%)
Annual neurosurgery volume (median [IQR])	400 [350; 700]
Number of neurosurgery beds (median [IQR])	63 [45; 116]
Reduction in neurosurgery case volume due to COVID-19	
< 25%	20 (13.9)
26–50%	56 (38.9)
51–75%	42 (29.2)
76–100%	26 (18.1)
Reduction in number of dedicated neurosurgery ORs	
Little to no reduction	66 (45.8)
Small reduction (< 1/3 of neurosurgery ORs)	6 (4.2)
Moderate reduction (< 2/3 of neurosurgery ORs)	13 (9.0)
Major reduction (≥ 2/3 of neurosurgery ORs)	19 (13.2)
No dedicated ORs (100% reduction of neurosurgery ORs)	9 (6.3)
Reduction in number of neurosurgery ICU beds	
Little to no reduction	93 (64.6)
Small reduction (< 1/3 of total ICU beds)	8 (5.6)
Moderate reduction (< 2/3 of total ICU beds)	11 (7.6)
Major reduction (≥ 2/3 of total ICU beds)	15 (10.4)
No dedicated ICU beds (100% reduction of ICU beds)	9 (6.3)
Current number of ventilators (median [IQR])	9 [4; 20]
Number of hospitals with increased ventilators	42 (29.2)
Percentage increase in number of ventilators (median)	0.5%
Level of personal protection by neurosurgeons operating on COVID-19-negative patients	
Routine gowning	73 (50.7)
Routine gowning with N95 mask	35 (24.3)
Gowns with junctions sealed, with air supply or self-contained breathing apparatus	21 (14.6)
Completely enclosed gowns with self-contained breathing apparatus	15 (10.4)
Scheduled neurosurgeries still being performed as planned on COVID-19 positive patients	34 (23.4)
At the peak of the pandemic, was there shortage of COVID-19 related supplies/PPE in the hospital?	
Little or no disruption	40 (27.8)
Moderate disruption	71 (49.3)
Severe disruption	20 (13.9)
Level of personal protection by neurosurgeons operating on COVID-19-positive patients	
Routine gowning	14 (9.7)
Routine gowning with N95 mask	17 (11.8)
Gowns with junctions sealed, with air supply or self-contained breathing apparatus	11 (7.6)
Completely enclosed gowns with self-contained breathing apparatus	101 (70.1)
Changes to pre-operative COVID-19 screening guidelines for CNS tumor patients	120 (83.3)
If yes, what changes?	
Confirmation of COVID-19 status by laboratory test as negative	58 (48.4)
Screening for COVID-19 based on history, clinical manifestation, and imaging	62 (51.6)
Suspension of post-operative adjuvant therapy	52 (36.1)
If no, what measures have been taken to administer post-operative adjuvant therapy?	
Home-based assisted treatment	4 (2.8)
Strict isolation, single room treatment	14 (9.7)
Intensive screening for COVID-19	29 (20.1)
Improving the protection level of doctors and patients	12 (8.3)
Referral to other hospitals	35 (24.3)
Staff training to operate ventilators?	93 (64.6)
Number of neurosurgeons/fellows per hospital (median [IQR])	16 (9; 30)
Personnel relocation from neurosurgery to other departments	91 (63.2)
Relocation of:	
Neurosurgeons	68 (47.2)
Operating room nurses/technicians	35 (24.3)
Trainees	75 (52.1)
ICU nurses	91 (63.2)
Floor nurses	91 (63.2)
Routine continuation of research	21 (14.6)
Suspension of research	85 (59.0)
Modifications of research	31 (21.5)
Research personnel working from home	14 (9.7)
Clinical research continuing but basic science research suspended	3 (2.1)
Segregation of research personnel	14 (9.7)
Trainees permitted to perform cases	67 (46.5)
Trainees required to stay at home unless on call or critically needed	78 (54.2)
Medical students mandated to stay at home	104 (72.2)

*CNS* central nervous system, *ICU* intensive care unit, *IQR* inter-quartile range, *OR* operating room, *SD* standard deviation

Nineteen hospitals (9.0%) reported > 66% reduction in their number of dedicated neurosurgery rooms and 9 (6.3%) reported a 100% reduction. Fifteen (10.4%) hospitals reported > 66% reduction and 9 (6.3%) reported a 100% reduction in their number of neurosurgery intensive care unit beds (Table 2).

Ninety-one (63.2%) hospitals reported moderate to severe disruption of COVID-19-related supplies and PPE in their hospitals. Seventy-three (50.7%) hospitals reported routine gowning by neurosurgeons operating on COVID-19 negative patients. Thirty-four (23.4%) still performed scheduled surgeries as planned in COVID-19 positive patients. Majority of hospitals (101 [70.1%]) described wearing completely enclosed gowns with self-contained breathing apparatus if operating on COVID-19 positive patients.

One-hundred and twenty (83.3%) hospitals described changes to pre-operative COVID-19 screening guidelines for CNS tumor patients. Fifty-two (36.1%) hospitals suspended post-operative adjuvant therapy and 94 (65.2%) instituted different measures to administer post-operative adjuvant therapy ranging from home-based assisted treatment in 4 (2.8%) hospitals to referral to other hospitals (35 [24.3%]).

### Impact of COVID-19 pandemic on neuro-oncological surgery personnel

The median number of neuro-oncological surgeons/fellows per responding hospital was 16 (9; 30). Ninety-three respondents (64.6%) trained additional staff to operate ventilators. Ninety-one (63.2%) reported a relocation of personnel from neurosurgery to other departments: 68 (47.2%) hospitals relocated neurosurgeons, 75 (52.1%) relocated trainees, 35 (24.3%) relocated operating room nurses/technicians, and 91 (63.2%) hospitals relocated ICU and ward nurses. (Fig. 1, Table 2).

### Impact of COVID-19 pandemic on neurosurgery education and research

Twenty-one (14.6%) hospitals reported routine continuation of neurosurgery research, 85 (59.0%) suspended all research, and 31 (21.5%) reported modifications to the conduct of research (Table 2). The suspension of clinical research by hospitals was not correlated (r = 0.30, *p* = 0.97) with the number of COVID-19 cases per million inhabitants in the respondent’s country/province.

Sixty-seven (46.5%) hospitals still allowed trainees to take part in performing neurosurgery cases, while 78 (54.2%) mandated trainees to stay at home unless on call or critically needed.

One-hundred and four (54.2%) hospitals mandated medical students to stay at home (Table 2).

### Impact of COVID-19 pandemic on hospital policies

The responding hospitals reported different screening criteria to determine patient eligibility for surgery: 3 (2.1%) hospitals discontinued all elective cases and emergency cases, while 97 [67.4%] hospitals continued elective neurosurgery in only COVID-19 negative patients (Table 3).

**Table 3:** Operational impact of Coronavirus disease 2019 (COVID-19) pandemic

Variable	Number of hospitals (%)
Criteria for performing neurosurgery	
Only emergency surgery in COVID-19-negative patients (diagnosed by imaging and laboratory examination)	5 (3.5)
Only emergency surgery in COVID-19-negative patients (diagnosed by clinical manifestation and imaging)	2 (1.4)
Only emergency surgery (regardless of COVID-19 status)	16 (11.1)
Neurosurgery in only COVID-19-negative patients (diagnosed by imaging and laboratory examination)	44 (30.6)
Neurosurgery in only COVID-19-negative patients (diagnosed by clinical manifestation and imaging)	53 (36.8)
Other criteria	15 (10.4)
Emergency neurosurgery status	
No emergency surgery	9 (6.3)
Only for COVID-negative patients	52 (36.1)
Emergency surgery performed routinely	83 (57.6)
Elective neuro-oncological surgery status	
No elective surgery	52 (36.1)
Only for COVID-negative patients	74 (51.4)
Elective surgery performed routinely	18 (12.5)
Protocols to restrict visitors to neurosurgery department	141 (97.9)
Discussion of ethical issues concerning how decisions will be made in the event healthcare resources must be prioritized and allocated	122 (84.7)
Patient follow-up for discharged patients	
In-person	10 (6.9)
Telehealth	27 (18.8)
Phone call	107 (74.3)
Mobile nurses	0 (0%)
Anticipation that the current COVID-19 pandemic will be contained in the upcoming weeks	124 (86.1)
Assessment of how soon operating restrictions might be lifted	
< 1 month	36 (25.0)
1–3 months	100 (69.4)
3–6 months	3 (2.1)
> 6 months	1 (0.7)
Anticipation of COVID-19-related disruption to neurosurgery department supplies	
Little or no disruption	61 (42.4)
Moderate disruption	70 (48.6)
Severe disruption	13 (9.0)
Anticipation of further COVID-19-related re-allocation of personnel from neurosurgery department	
Little or none	116 (80.6)
Moderate	26 (18.1)
Extensive	2 (1.4)

Ten (6.9%) hospitals performed in-person patient follow-up, 27 (18.8%) were using telehealth, and 107 (74.3%) were using phone calls (Table 3).

### Future directions

A total of 124 (86.1%) respondents anticipated that the current COVID-19 pandemic will be contained during the upcoming weeks. 36 (25.0%) respondents anticipated that the restrictions preventing full neuro-oncological surgery activity will be lifted in < 1 month, 100 (69.1%) in 1–3 months, 3(2.1%) in 3–6 months, and 1 (0.7%) in > 6 months.

Eighty-three (57.6%) hospitals anticipated moderate-to-severe further COVID-19 related disruption to their neurosurgical supplies; thirty (19.5%) respondents anticipated moderate-to-extensive further COVID-19 related relocation of personnel from their neurosurgery hospitals (Table 3).

## Discussion

Our survey of 144 neurosurgery hospitals and 3699 neurosurgeons provides a comprehensive perspective on the current neuro-oncological surgery response to the COVID-19 pandemic. Hospitals’ responses varied according to their resources and the phases of the pandemic in their respective geographic locations. Majority of the respondents were in phase 5/6 (sustained or widespread human infection) and post-peak phase of the pandemic according on the World Health Organization classification (Additional file 1: Figure S1) [10]. Most hospitals reported 26–50% reduction in neurosurgery case volume and over one-third of the responding hospitals completely suspended elective neurosurgery and post-operative adjuvant therapy; over 70% of the respondents anticipate that current neurosurgery restrictions will still continue to last for more than one month.

Our results highlight that in addition to careful planning for effective patient triage, the strategies to minimize the immediate and long-term impact of any pandemic on a neuro-oncological surgery setting must comprise overall expansion of the healthcare system’s capacity, safety measures for healthcare providers, and detailed policies to resume routine surgical activity against the backlog of long patient waiting lists.

### Patient triage

The responding hospitals reported a median reduction of 26–50% in neurosurgery case-volume and over 80% of respondents modified pre-operative COVID-19 screening guidelines for CNS tumor patients [6, 8, 11]. The implications of delaying neuro-oncological surgery for patients requiring treatment for several weeks are reported in literature and longer wait time from glioblastoma presentation to surgery is a risk factor for developing additional symptoms which causes patients to lose their favorable prognosis [12]. The COVID-19 pandemic has produced a silent sub-epidemic of people who need care at hospitals but are reluctant to come in. In neuro-oncological surgery, patients with CNS tumors face additional challenges due to their immunocompromised status and complexity of surgery and delaying treatment and can be dangerous.

Our survey demonstrates that despite majority of respondents being in advanced and post-peak phases of the pandemic, neuro-oncological surgery remains far from routine. Considering the chronic impact of such a global pandemic, strategies to triage patients for neuro-oncological surgery must consider the long-term effects of delaying surgery and balance the urgency of patients’ presentation (increased intracranial pressure and cerebral hernia, neurological dysfunction, tumor stroke, etc.) with the need to reallocate hospital resources in a pandemic situation. The American College of Surgeons recommends that elective oncological surgery cases are cancelled or postponed during the COVID-19 pandemic [13]. Although helpful, these recommendations do not address the complexity of triaging urgent neuro-oncological surgery cases and waiting list patients with delayed presentation. For asymptomatic patients with low grade or benign gliomas, elective surgery must be postponed until a safer time. However, for patients with malignant tumors such as high-grade gliomas or benign tumor with severe symptoms, surgery must be promptly scheduled since delay in surgery may reduce the favorability of prognosis. In emergency cases, such as patients with acute hydrocephalus or cerebral herniation, surgery must be arranged emergently. In these instances, the results of COVID-19 testing may not be available before surgery, and surgery should be carried out under strict precautions to minimize possible exposure [8].

Due to the complexity of CNS tumors, in addition to careful attention to patient risk factors such as presenting symptoms and pathology, effective triage must involve optimizing medical therapy with frequent follow-up [14]. This is challenging and must be led by a team of specialists in neuro-oncological surgery at every institution. It may be reasonable to perform very complex neuro-oncological surgeries in the early phases of a pandemic when long-term resources are available. In times of worsening pandemic phases, however, the ethics of this may be less justifiable. As part of effective patient triage, COVID-19 screening should be performed in all patients due to the asymptomatic presentation of the disease. While current data is limited, based on our experience and other anecdotal evidence, patients with COVID-19 are associated with significant morbidity and mortality during their perioperative course and, therefore, where possible, neuro-oncological surgery should be delayed until patients are disease-free. In patients with acute presentation where neuro-oncological surgery cannot be deferred supportive therapy and less-invasive interventions may be more suitable where possible.

Similar strategies have been adopted elsewhere to tackle neuro-oncological surgery in the early and late phases of the pandemic [15–18]. In a single-center study from Texas, top surgical priority patients are those with large masses, progressive neurologic decline, severe pain, no nonsurgical options, or when diagnosis via surgery is required to initiate therapy [19]. Another study recommended using the Delphi method by an expert panel to assess the urgency of different neurosurgical interventions [20]. Bajunaid et al. from the Saudi Arabian Neurosurgical Association recommended surgery for all intracranial tumors affecting consciousness, causing hemodynamic instability, or acute vision loss due to optic nerve/chiasm compression [16]. The Society of British Neurological Surgeons also recommends using day-case surgery where possible and short-stay overnight surgery for all other routine oncology procedures without admission to critical care. In addition, they recommend resection of malignant glioma in patients suitable for adjuvant oncology treatment, posterior fossa tumors (malignant or non-malignant) causing symptomatic or life-threatening hydrocephalus, meningioma causing major mass effect, and supratentorial symptomatic brain metastases. For rare brain tumors causing hydrocephalus, temporizing measures such as endoscopic third ventriculostomy or ventriculoperitoneal shunt and delaying definitive surgery were recommended (exceptions are germ cell tumors and pineoblastoma) [21].

### Expanding capacity and pre-planning

Although COVID-19 patients accounted for fewer than 10% of hospital inpatients at hospitals surveyed, over one-third of hospitals reported reduction in the intensive care beds available for neuro-oncological surgery patients, reflective of the prolonged ventilator dependence associated with COVID-19 hospitalized patients [22]. Alarmingly, only 29% of hospitals had expanded their number of ventilators by a median of 0.5% only. In over two-thirds of hospitals, neuro-oncological surgery personnel were reallocated to other services.

Most hospitals keep their running cost low through short-term supplies. During the early phases of an escalating pandemic, essential equipment and medication should be stocked in anticipation of worsening crisis. Policies should mandate conservation of PPE from the onset and healthcare personnel should be re-educated on the use of PPE and management of infected patients. Telemedicine and other remote follow-up approaches should be facilitated and new treatment algorithms to minimize duration of hospital stay and favor minimally invasive procedures should be planned through inter-departmental expert consensus.

It should also be emphasized that such policies must be fluid allowing modifications in response to worsening crisis. The indications for urgent/emergent surgery at the height of a pandemic will be different from those in the early- and post-peak pandemic phases based on the availability of critical care resources. Furthermore, based on hospital logistics, thorough plans should be developed for handling post-operative complications, especially in COVID-19 CNS tumor patients undergoing neuro-oncological surgery. The gradual resumption of routine practice following plateauing in pandemic related-admissions should also be planned with high-risk patients prioritized for early surgery.

### Protecting the workforce

Patients admitted for neuro-oncological surgery must be carefully screened for COVID-19 to minimize exposure to operating personnel from aerosolization during intubation, extubation, and disconnection of ventilators. Majority of the respondents reported performing neuro-oncological surgery in only COVID-19 negative patients and strict personal protection when operating on COVID-19 patients. This has been emphasized by a joint statement by the American Society of Anesthesiologists which recommend staff to be in powered air-purifying respirators during aerosol generating procedures to minimize operating room outbreak. [23] Current Chinese guidelines for aerosol transmissible diseases mandate precautions for healthcare professional in 3 tiers: level 1 precautions require the use of a surgical cap, surgical face mask, protective gown and gloves while Level 3 precautions mandate surgical cap, N95 face mask, goggles, faceshield, full face piece respirator, protective gown, gloves.

As majority of the hospitals in our survey responded, all patients admitted for surgery must undergo COVID-19 screening, including contact tracing, clinical evaluation, novel coronavirus nucleic acid and antibody test, and chest CT scan. Further precautions will be dictated by results of pre-procedural screening. In low risk areas, patients confirmed to be COVID-19 negative could be operated under level 1 precautions. In high-risk areas, or for patients suspected of being infected and requiring emergency neuro-oncological surgery, it is important that the tertiary health care facility is well equipped to perform the surgery. Where possible, COVID-19 positive should be referred to specified regional hospitals accepting infected patients for neuro-oncological surgery. For emergency cases however, surgery must be carried out under level 3 protection. It is essential that a negative pressure operating room is available for the management of these patients. It must be recognized, however, that majority of the hospitals globally currently have limited supply of N95 masks which may not be sufficient for the entire operating team. Consequently, it is recommended that all personnel not wearing N95 masks leave the operating room during potential aerosol-generating steps of surgery. [24, 25] Other measures to minimize aerosol generation during neurosurgery include lower coagulation settings for electrocautery, approaches that reduce the risk of infection, such as endoscopic transsphenoidal surgery, avoidance of endonasal procedures due to the very high risk of infection due to aerosol generation [26, 27].

Hospitals should further expand testing capacity to efficiently diagnose and isolate exposed surgeons and healthcare personnel. This may explain why the most common response when respondents were asked how the long-term impact of the COVID-19 pandemic could be minimized was effective implementation of “guidelines and protocol developed by infectious disease specialists to protect staff”. In the absence of reliable equipment and advice, neuro-oncological surgery hospitals adopted the few containment strategies over which they had control, with 98% restricting access to visitors and three-quarters performing outpatient follow-up remotely.

### Research and education

Research activity was suspended or reduced in 59% of responding hospitals which may obstruct efforts to obtain data and highlights the need for a version of crowd-sourcing to assemble and disseminate relevant data that could inform practice in a timely manner. Educational activity was similarly impacted which may have greater impact in smaller academic institutions with limited surgical neuro-oncology case volume where exposure to neurosurgery training may be curtailed. This could explain why 46% were still allowing trainees to operate, even at the expense of infection exposure and prolongation of surgery.

### Post-peak phase of the pandemic

**Fig. 2:**
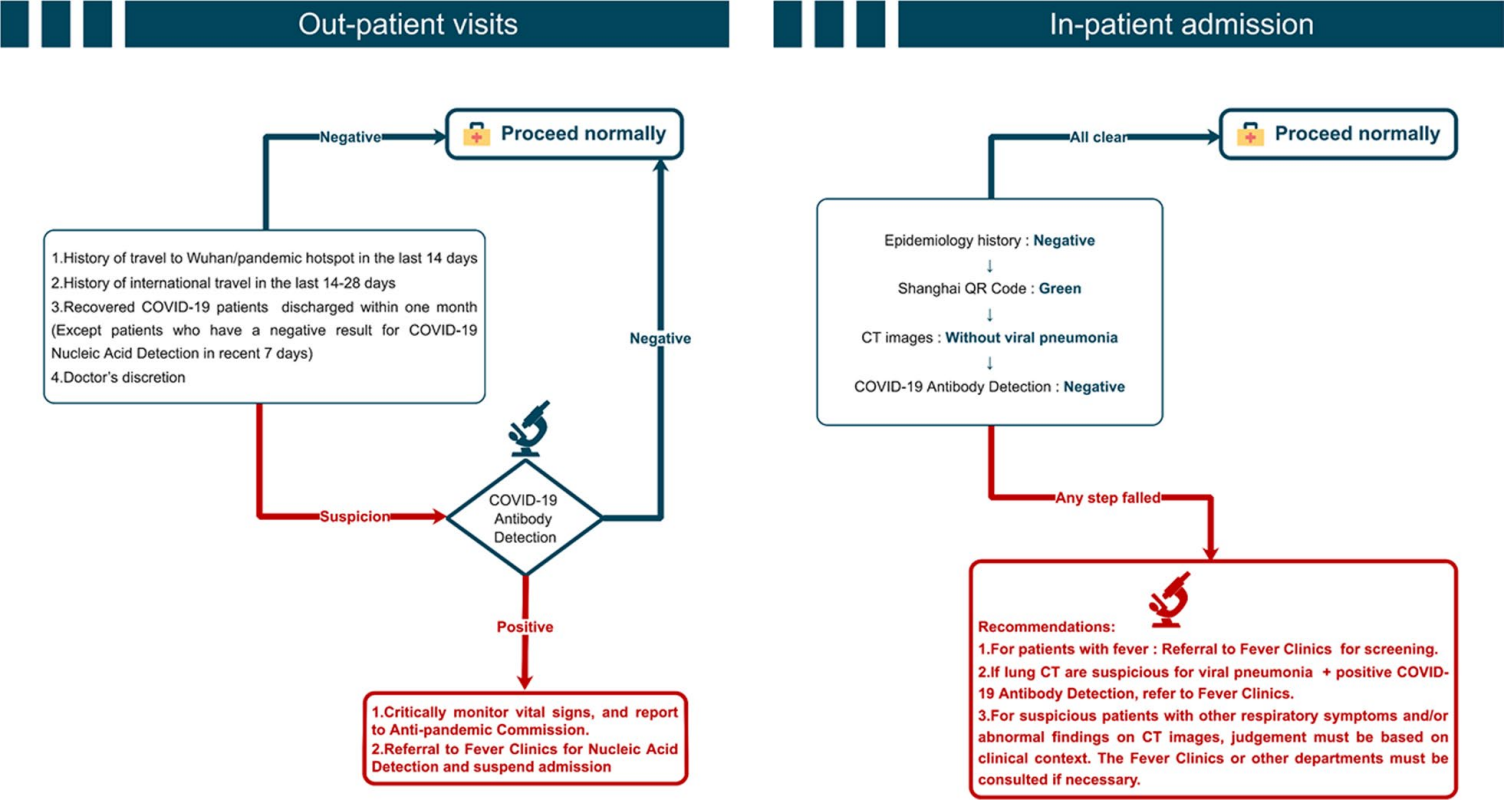
Huashan Hospital’s algorithm for screening patients for neuro-oncological surgery in the post-peak phase pandemic. **a** Algorithm for out-patient clinic visit patients; **b** Algorithm for in-patient admission

Reports on neurosurgery response to COVID-19 have mostly addressed the acute response to the pandemic. There is uncertainty on how to mend neurosurgical activity in the post-peak phases and immediate return to routine neurosurgical activity is not possible. With continued risk of pandemic rebound, resumption of surgery for patients on waiting lists must be balanced against careful screening of all admitted patients. To accommodate this, in Shanghai Huashan Hospital, new screening algorithms have been developed to resume neuro-oncological surgical activity to full capacity (Fig. 2). All planned admissions (clinic or emergency) are screened for COVID-19 with particular attention to patients coming from pandemic hotspots and other countries. Patients can also be recommended for testing based on their neuro-oncological surgeon’s discretion.

### Limitations

The main limitation is that this study is a snapshot of a rapidly evolving situation, affecting very heterogeneous populations with wide variation in impact and response. Although survey participants were drawn from academic- and non-academic neuro-oncological surgery hospitals of different sizes, respondents were limited to Asian hospitals and findings may not be fully reflective of other neurosurgery hospitals in other countries. Furthermore, hospitals dedicated to surgical neuro-oncology, and without emergency rooms will have different priorities, as will those neurosurgery hospitals that have been turned over completely to COVID-19 care as part of a planned regional response.

## Conclusions

Majority of the respondents to our survey reported reduced neuro-oncological surgery activity, hospital policies, personnel reallocation, and curtailment of educational and research activities in response to the COVID-19 pandemic. The persistent widespread interruption of neuro-oncological surgery in even post-peak phases of the pandemic raises serious concerns about the long-term impact of the pandemic on surgical neuro-oncology patients and highlights the essence of timely measures for pandemic preparedness, patient triage, and workforce protection.

## Supplementary Information

**Additional file 1.** Supplementary File.

## Data Availability

Requests for data sharing will be considered by the authors upon reasonable request.

## Abbreviations

CNS: Central nervous system
COVID-19: Coronavirus disease 2019
CSNO: Chinese Society of Neuro-Oncology
ICU: Intensive care unit
PPE: Personal protective equipment
SD: Standard deviation
