# Supplementary material for: Impact of a pandemic on surgical neuro-oncology—maintaining functionality in the early phase of crisis
Source: BMC Surg. 2021 Jan 18;21:40. doi: 10.1186/s12893-021-01055-z (PMC7812331; doi:10.1186/s12893-021-01055-z)
Supplement: Supplementary file 1 — Additional file 1. Supplementary File. [file 12893_2021_1055_MOESM1_ESM.docx]

**SUPPLEMENTARY APPENDIX**

**Supplementary Table 1.** Full questionnaire distributed to neurosurgery hospitals.

| ***Details of Site***  **Name of Hospital:**  **Total number of hospital beds:**  **Hospital Location**   - City: - Country:   Annual neurosurgery case volume:  Number of neurosurgery beds:  ***Current COVID-19 status***  **When was the first case of COVID-19 diagnosed in your country? (if you know)**  ☐December, 2019  ☐January, 2020  ☐February, 2020  ☐March, 2020  **What is the current number of COVID-19 infections in your country? (Optional)**  ☐0-100  ☐100-500  ☐500-1000  ☐>1000  Number:  **What is the total number of COVID-19 deaths in your country to date? (Optional)**  ☐ 0-100  ☐ 100-500  ☐ 500-1000  ☐>1000  Number:  **How severe are the public health restrictions in place to control COVID-19 spread?**  ☐None  ☐Mild (Restriction of social gatherings of more than 100 people)  ☐Moderate (Restriction of social gatherings of more than 10 people)  ☐Severe (Ban on all social gatherings but permission for certain businesses to function)  ☐Total curfew/lockdown with only emergency services active  **Is your hospital a COVID-19 dedicated center?**  ☐Yes  ☐No  **Have patients with COVID-19 been diagnosed at your hospital?**  ☐Yes  ☐No  **What is the total number of COVID-19 patients currently hospitalized at your hospital?**  ☐0-10  ☐11-50  ☐50-100  ☐>100  Number:  ***Neurosurgery - Logistics***  **What has been the % reduction in neurosurgery case volume at your hospital?**  ☐0-25%  ☐25-50%  ☐50-75%  ☐75-100%  **What is your neurosurgery subspecialty? (E.g. General neurosurgery, Neurooncology, Cerebrovascular, etc.)**  **………………………………..**  **Has your department suspended scheduling of elective neuro-oncological surgeries?**  ☐Suspended all elective surgeries  ☐Surgeries permitted on COVID-19 negative patients  ☐No restrictions on elective surgeries  **Has your department suspended scheduling of emergency surgeries?**  ☐Suspended all emergency surgeries  ☐Surgeries permitted on COVID-19 negative patients  ☐No restrictions on emergency surgeries  **What are your current screening criteria to determine patient eligibility for neurosurgery in the presence of restrictions?**  ☐ **Criteria …………….**  ☐ **None**  **What is the number of dedicated neurosurgery operating rooms in your center before the COVID-19 pandemic and currently?**   - Before COVID-19: - Currently:   **What is the number of dedicated neurosurgery ICU beds at your center before the COVID-19 pandemic and currently?**   - Before COVID-19: - Currently:   **Does your hospital test COVID-19 before neurosurgery?**  ☐ Routine test  ☐ When patient be suspected  ☐ Never  **What level of personal protection do neurosurgeons take for surgical patients who have tested negative for COVID-19 before surgery?**  ☐ Routine gowning  ☐Level I (Routine gown with respirator or N95 mask)  ☐Level II (Suit with junctions sealed, supplied air or self-contained breathing apparatus)  ☐Level III (Completely sealed suit and self-contained breathing apparatus)  **Is scheduled surgery still performed as planned on surgery patients who test positive for COVID-19 before surgery?**  ☐Yes  ☐No  **What level of personal protection do surgeons take for surgery patients who have tested positive COVID-19 before surgery?**  ☐ Routine gowning  ☐Level I (Routine gown with respirator or N95 mask)  ☐Level II (Suit with junctions sealed, supplied air or self-contained breathing apparatus)  ☐Level III (Completely sealed suit and self-contained breathing apparatus)  **Has preoperative screening for brain tumor patients changed during the outbreak?**  ☐Yes  ☐No  **If yes, what preoperative screening procedures are currently in place?**  **Has postoperative adjuvant therapy for brain tumor patients been suspended?**  ☐Yes  ☐No  **If no, what measures have been taken to ensure adequate administration of adjuvant therapy to patients?...............**  **What is the total number of ventilators, which are currently available and staffed in your hospital? (if you know)………………**  **Has the number of ventilators been increased? (if you know)**  ☐Yes  ☐No  **24. Are more staff being trained to operate ECMO machines?**  ☐Yes  ☐No  ***Neurosurgery – Personnel***  **What is the total number of neurosurgeons/fellows at your center?**  **Have personnel been relocated to treatment of COVID-19 patients?**  ☐ Yes  ☐ No  **If yes, how many personnel (%) have been relocated to:**   - ICU: - Wards: - Screening: - Other roles:   **What personnel have been relocated?**  ☐Neurosurgeons  ☐Trainees  ☐Operating room nurses/technicians  ☐ICU nurses  ☐Floor nurses  ***Neurosurgery education research***  **Has neurosurgery research been suspended?**  ☐ Yes  ☐ No  **Are trainees allowed to operate?**  ☐ Yes  ☐ No  **Have medical students been requested to stay home?**  ☐ Yes  ☐ No  **What modifications have implemented for clinical and basic science research?**  **…………………………….**  ***Departmental policies***  **Have criteria and protocols been developed for limiting or restricting visitors to the neurosurgery department?**  ☐ Yes  ☐ No  **Have ethical issues been discussed concerning how decisions will be made in the event healthcare services must be prioritized and allocated (e.g., decisions based on probability of survival)?**  ☐ Yes  ☐ No  **How is follow-up being performed for discharged patients?**  ☐In-person  ☐Telehealth  ☐Phone call  ☐Mobile nurses  ***Future direction***  **Based on the current public health policy of your country, are you anticipating the current COVID-19 pandemic in your country to be contained over the upcoming weeks?**  ☐ Yes  ☐ No  **If yes, how soon might operating restrictions be lifted in your opinion?**  ☐<1 month  ☐1-3 months  ☐3-6 months  ☐>6 months  **Are you anticipating significant COVID-19-related disruption of hospital supplies to your neurosurgery department should the pandemic worsen?**  ☐Little or no disruption  ☐Moderate disruption  ☐Severe disruption  **Are you anticipating further COVID-19-related re-allocation of personnel from your neurosurgery department?**  ☐Little or none  ☐Moderate  ☐Extensive  **How can the long-term impact of the COVID-19 pandemic on neurosurgery procedures at your hospital be minimized?** |
| --- |

**Supplementary Table 2.** Location of responding centers and prevalence of Coronavirus disease 2019 (COVID-19) as of April 20, 2020.

| Variable | Number of centers (%) | Number of reported COVID-19 cases per country/province | Total country/province population | Number of reported COVID-19 cases per million inhabitants |
| --- | --- | --- | --- | --- |
| Overall | 144(100%) |  |  | - |
| Location of center |  |  |  |  |
| China | 134 (93.1%) | 84250 | 1332810869 | 63.2 |
| - *Anhui* | 8 (5.6%) | 991 | 59,500,468 | 16.7 |
| - *Beijing* | 6 (4.2%) | 593 | 19,612,368 | 30.2 |
| - *Fujian* | 5 (3.5%) | 355 | 36,894,217 | 9.6 |
| - *Gansu* | 3 (2.1%) | 139 | 25,575,263 | 5.4 |
| - *Guangdong* | 13 (9%) | 1582 | 104,320,459 | 15.2 |
| - *Guangxi* | 5 (3.5%) | 254 | 46,023,761 | 5.5 |
| - *Guizhou* | 3 (2.1%) | 147 | 34,748,556 | 4.2 |
| - *Hainan* | 3 (2.1%) | 168 | 8,671,485 | 19.4 |
| - *Hebei* | 5 (3.5%) | 328 | 71,854,210 | 4.6 |
| - *Henan* | 5 (3.5%) | 1276 | 94,029,939 | 13.6 |
| - *Heilongjiang* | 2 (1.4%) | 913 | 38,313,991 | 23.8 |
| - *Hubei* | 19 (13.2%) | 68128 | 57,237,727 | 1190.3 |
| - *Jilin* | 3 (2.1%) | 106 | 27,452,815 | 3.9 |
| - *Jiangsu* | 9 (6.3%) | 653 | 78,660,941 | 8.3 |
| - *Jiangxi* | 2 (1.4%) | 937 | 44,567,797 | 21.0 |
| - *Liaoning* | 2 (1.4%) | 146 | 43,746,323 | 3.3 |
| - *Inner Mongolia* | 3 (2.1%) | 194 | 24,706,291 | 7.9 |
| - *Ningxia* | 4 (2.8%) | 75 | 6,301,350 | 11.9 |
| - *Qinghai* | 1 (0.7%) | 18 | 5,626,723 | 3.2 |
| - *Shandong* | 2 (1.4%) | 787 | 95,792,719 | 8.2 |
| - *Shanxi* | 3 (2.1%) | 197 | 35,712,101 | 5.5 |
| - *Shaanxi* | 4 (2.8%) | 277 | 37,327,379 | 7.4 |
| - *Shanghai* | 3 (2.1%) | 638 | 23,019,196 | 27.7 |
| - *Sichuan* | 5 (3.5%) | 561 | 80,417,528 | 7.0 |
| - *Tianjin* | 3 (2.1%) | 189 | 12,938,693 | 14.6 |
| - *Tibet* | 1 (0.7%) | 1 | 3,002,165 | 0.3 |
| - *Xinjiang* | 3 (2.1%) | 76 | 21,815,815 | 3.5 |
| - *Yunnan* | 1 (0.7%) | 184 | 45,966,766 | 4.0 |
| - *Zhejiang* | 3 (2.1%) | 1268 | 54,426,891 | 23.3 |
| - *Chongqing* | 3 (2.1%) | 579 | 28,846,170 | 20.1 |
| - *Hong Kong* | 2 (1.4%) | 1025 | 7,500,700 | 136.7 |
| India | 1 (0.7%) | 17615 | 1,324,000,000 | 13.3 |
| Japan | 6 (4.2%) | 10797 | 124,776,364 | 86.5 |
| South Korea | 3 (2.1%) | 10674 | 51,635,256 | 206.7 |

**Supplementary Table 3.** List of responding centers.

| Number | Name of Hospital | Country |
| --- | --- | --- |
| 1 | 940 Hospital of the Support Force | China |
| 2 | Affiliated Hospital of Guilin Medical University | China |
| 3 | Ankang Central Hospital | China |
| 4 | AoHanQi Hospital | China |
| 5 | Beijing Tiantan Hospital | China |
| 6 | Bengbu First people's Hospital | China |
| 7 | Bijie First People's Hospital | China |
| 8 | Cancer Center of Guangzhou Medical University | China |
| 9 | Sun Yat-sen University Cancer Center | China |
| 10 | Cancer Hospital Chinese Academy of Medical Science | China |
| 11 | Guangdong Province People’s Hospital | China |
| 12 | Chongqing University Cancer Hospital | China |
| 13 | Dengfeng People's Hospital | China |
| 14 | Ezhou Central Hospital | China |
| 15 | First Hospital of Shanxi Medical University | China |
| 16 | Fujian Cancer Hospital | China |
| 17 | Fujian Medical University Union Hospital | China |
| 18 | Gaoyi County Hospital | China |
| 19 | General Hospital of Ningxia Medical University | China |
| 20 | General Hospital of Southern Theatre Command | China |
| 21 | Guangdong Agricultural Reclamation Center Hospital | China |
| 22 | Guangdong Traditional Chinese Medicine Hospital | China |
| 23 | Guangxi Medical University Affiliated Tumor Hospital | China |
| 24 | Guizhou Provincial People's Hospital | China |
| 25 | Guyuan People's Hospital | China |
| 26 | Hainan Cancer Hospital | China |
| 27 | Hainan General Hospital | China |
| 28 | Harbin Medical University Cancer Hospital | China |
| 29 | Hefei Eighth People's Hospital | China |
| 30 | Hefei Second People's Hospital | China |
| 31 | Henan Cancer Hospital | China |
| 32 | Henan Provincial People's Hospital | China |
| 33 | Huaibei miner General Hospital | China |
| 34 | Huaihe Hospital of Henan University | China |
| 35 | Hualong Hospital | China |
| 36 | Huangshi Aikang Hospital | China |
| 37 | Huangshi Central Hospital | China |
| 38 | Huashan Hospital | China |
| 39 | Hubei Cancer Hospital | China |
| 40 | Hubei Provincial Hospital of Integrated Traditonal and Western Medicine | China |
| 41 | Huhhot First Hospital | China |
| 42 | Inner Mongolia People's Hospital | China |
| 43 | Ji'an Central Hospital | China |
| 44 | Jiangsu Cancer Hospital | China |
| 45 | Jilin Cancer Hospital | China |
| 46 | Jingmen First People's Hospital | China |
| 47 | Jingzhou First People's Hospital | China |
| 48 | Jiujiang First People's Hospital | China |
| 49 | Lanzhou University Second Hospital | China |
| 50 | Liaocheng People's Hospital | China |
| 51 | Liaoning Cancer Hospital | China |
| 52 | Luotian People's Hospital | China |
| 53 | Macheng People's Hospital | China |
| 54 | Zhongda Hospital | China |
| 55 | Minda Hospital of Hubei Minzu University | China |
| 56 | NO.215 Hospital of Shaanxi Nuclear Industry | China |
| 57 | Panzhihua Central Hospital | China |
| 58 | Peking Union Medical College Hospital | China |
| 59 | People's Hospital of Xinjiang Autonomous Region | China |
| 60 | Qilu Hospital of Shandong University | China |
| 61 | Qinghai Provincial People's Hospital | China |
| 62 | Qingyuan People's Hospital | China |
| 63 | Red Cross Hospital of Yulin City | China |
| 64 | Renmin Hospital of Wuhan University | China |
| 65 | Sanbo Brain Hospital Capital Medical University | China |
| 66 | Sanbofuneng Brain Hospital | China |
| 67 | Shanghai Gamma Knife Hospital | China |
| 68 | Shanghai Pudong Hospital | China |
| 69 | Shanxi Hospital of Integrated Traditonal and Western Medicine | China |
| 70 | Shanxi Provincial People's Hospital | China |
| 71 | Shayang People's Hospital | China |
| 72 | Shenzhen Second People's Hospital | China |
| 73 | Shenzhen Third People's Hospital | China |
| 74 | Sichuan Cancer Hospital | China |
| 75 | Sichuan Provincial People's Hospital Friendship Hospital | China |
| 76 | Siping Central Hospital | China |
| 77 | Subei People's Hospital | China |
| 78 | Sun Yat Sen Memorial Hospital of Sun Yat sen University | China |
| 79 | Suzhou Ninth People's Hospital | China |
| 80 | Taizhou Cancer Hospital | China |
| 81 | Tangshan Gongren Hospital | China |
| 82 | The Affiliated Hospital of Southwest Medical University | China |
| 83 | The Affiliated Hospital of Xuzhou Medical University | China |
| 84 | The Central Hospital of Wuhan | China |
| 85 | The First Affiliated Hospital of Anhui Medical University | China |
| 86 | The First Affiliated Hospital of China University of science and technology | China |
| 87 | The First Affiliated Hospital of Chongqing Medical University | China |
| 88 | The First Affiliated Hospital of Fujian Medical University | China |
| 89 | The First Affiliated Hospital of Guangxi Medical University | China |
| 90 | The First Affiliated Hospital of Hainan Medical University | China |
| 91 | The First Affiliated Hospital of Harbin Medical University | China |
| 92 | The First Affiliated Hospital of the Fourth Military Medical University | China |
| 93 | The First Affiliated Hospital of Xinjiang Medical University | China |
| 94 | The First Affiliated Hospital of Zhengzhou University | China |
| 95 | The First Bethune Hospital of Jilin University | China |
| 96 | The First People's Hospital of Lianyungang | China |
| 97 | The Fourth Hospital of Hebei Medical University | China |
| 98 | The Second Affiliated Hospital of Suzhou University | China |
| 99 | The Second Affiliated Hospital of Wannan Medical College | China |
| 100 | The Second Affiliated Hospital of Zhejiang University of Medicine | China |
| 101 | The Second Hospital of Dalian Medical University | China |
| 102 | The Second Hospital of Hebei Medical University | China |
| 103 | The Second People's Hospital of Guiyang | China |
| 104 | The Second People's Hospital of Tibet Autonomous Region | China |
| 105 | The Third Hospital of Hebei Medical University | China |
| 106 | Tianjin Hospital | China |
| 107 | Tianjin Huanhu Hospital | China |
| 108 | Tianjin Medical University Cancer Hospital | China |
| 109 | Tianshui combine traditional Chinese and Western Medicine Hospital | China |
| 110 | Tongling People's Hospital | China |
| 111 | Tsinghua University Affiliated Beijing Changgung Hospital | China |
| 112 | Union Shenzhen Hospital(Nanshan Hospital) | China |
| 113 | West China Hospital | China |
| 114 | Wuhan Changjiang Shipping General Hospital | China |
| 115 | Wuhan Puren Hospital | China |
| 116 | Wuhan Union Hospital | China |
| 117 | Wuxi Xishan People's Hospital | China |
| 118 | Wuzhong People's Hospital | China |
| 119 | Wuzhou Red Cross Hospital | China |
| 120 | Xiamen Chang Gung Hospital | China |
| 121 | Xiantao First People's Hospital | China |
| 122 | Xiji County People's Hospital | China |
| 123 | Xinjiang Children's Hospital | China |
| 124 | Xinqiao Hospital | China |
| 125 | Xuanwu Hospital Capital Medical University | China |
| 126 | Yanan University Affiliated Hospital | China |
| 127 | Yancheng NO.1 People's Hospital | China |
| 128 | Yunnan Cancer Hospital | China |
| 129 | Zhejiang Tumor Hospital | China |
| 130 | Zhongnan Hospital of Wuhan University | China |
| 131 | Zhujiang Hospital of Southern Medical University | China |
| 132 | National Cancer Center Korea | South Korea |
| 133 | Apollo Proton Cancer Centre | India |
| 134 | Queen Mary | China |
| 135 | Seoul National University Bundang Hospital | South Korea |
| 136 | Prince of Wales Hospital | China |
| 137 | Korea Univ Hospital | South Korea |
| 138 | Guangzhou First Municipal Peoples Hospital | China |
| 139 | Saitama Medical University International Medical Center | Japan |
| 140 | Yokohama City University | Japan |
| 141 | University Hospital Kyoto Prefectural University of Medicine | Japan |
| 142 | Niigata University Medical and Dental Hospital | Japan |
| 143 | Kobe Hospital | Japan |
| 144 | Kyoto Perfectual Graduate University of Medicine | Japan |
